# Breast cancer pharmacotherapy and imaging interpretation: pathophysiological perspectives and clinical application

**DOI:** 10.1007/s11604-025-01926-y

**Published:** 2025-12-24

**Authors:** Roka Namoto Matsubayashi, Nobutaka Iwakuma

**Affiliations:** 1 Breast Care Center and Clinical Research Institute, National Hospital Organization Kyushu Medical Center, Fukuoka, Japan; 2 Breast Surgery, Breast Care Center and Clinical Research Institute, National Hospital Organization Kyushu Medical Center, Fukuoka, Japan

**Keywords:** Breast cancer, Pharmacotherapy, Imaging, Microenvironment

## Abstract

Modern breast cancer treatment has evolved significantly, integrating advanced imaging techniques and a variety of drug therapies, including endocrine therapy, chemotherapy, molecularly targeted drugs, and immune checkpoint inhibitors. This study discusses the interplay of drug treatments and imaging diagnostics in optimizing patient outcomes. Key findings emphasize the importance of personalized treatments based on intrinsic subtypes and imaging-guided decisions, as well as the management of treatment-related adverse effects.

## Introduction

Breast cancer remains one of the most prevalent malignancies worldwide, with significant advancements in its management [1]. The introduction of molecularly targeted therapies and immune checkpoint inhibitors has improved survival rates and quality of life [1]. Imaging plays a crucial role in diagnosing, staging, and monitoring therapy effectiveness. This paper focuses on integrating imaging diagnostics with modern pharmacological approaches to achieve personalized treatment.

## Methodology

The data presented in this study includes insights from international clinical trials, case studies, and imaging-based evaluations. Imaging modalities such as mammography, ultrasound, computed tomography (CT), magnetic resonance imaging (MRI), and positron emission tomography (PET) are analyzed alongside drug efficacy in specific breast cancer subtypes: Luminal, HER2-positive, and Triple Negative (TNBC) [2, 3].

## Results and discussions

### Novel therapeutic strategies and tumor microenvironment

#### Postoperative adjuvant therapy with cyclin-dependent kinase 4/6 (CDK4/6) inhibitors (abemaciclib)

CDK4/6 inhibitors, such as abemaciclib, have become an important option for postoperative adjuvant therapy, particularly in patients with high-risk recurrence and positive lymph node metastasis. The MonarchE trial demonstrated the efficacy of abemaciclib in extending invasive disease-free survival (iDFS) in patients with lymph node-positive breast cancer. A total of 5,637 patients were enrolled, and abemaciclib, in combination with hormonal therapy, significantly reduced the risk of recurrence, especially in high-risk patients [4–6].

#### Comparison of adjuvant CDK4/6 inhibitors and S-1 therapy in HR-positive, HER2-negative breast cancer: patient selection, risk stratification, and outcomes

Adjuvant strategies for hormone receptor-positive (HR+), HER2-negative early breast cancer have diversified with the introduction of targeted agents designed to improve outcomes beyond standard endocrine therapy. Two pivotal randomized trials—MonarchE (CDK4/6 inhibitor abemaciclib) and POTENT (oral fluoropyrimidine S-1)—have demonstrated enhanced invasive disease-free survival (iDFS). However, differences in trial design, risk stratification, and patient demographics necessitate nuanced interpretation.

### CDK4/6 inhibitor Arm: the MonarchE trial

The MonarchE trial (N = 5,637) enrolled patients with high-risk early breast cancer, defined by:

-  ≥ 4 positive axillary lymph nodes (ALNs), or
- 1–3 ALNs plus tumor ≥ 5 cm, grade 3 histology, or Ki 67 ≥ 20% [4].

Participants received standard endocrine therapy with or without abemaciclib (150 mg BID for 2 years). At a median follow-up of ~ 2 years (interim), abemaciclib significantly improved 2-year iDFS (92.2% vs. 88.7%; HR 0.75, p = 0.01) [4]. Updated analyses at 54 months reaffirm sustained benefits in iDFS and distant relapse-free survival [5]. Common grade ≥ 3 adverse events included neutropenia (~ 20.6%) and diarrhea (~ 8.4%) [4].

### S-1 arm: the POTENT trial

The POTENT trial (N = 1,930) was a Japanese, open-label, phase III study that enrolled intermediate-to-high-risk patients:

- Node-positive or
- Node-negative with additional risk factors (e.g., tumor > 2 cm, high grade, vascular invasion) [7].

Patients received endocrine therapy ± S-1 (80–120 mg/day, 2 weeks on/1 week off for 12 months). At a median follow-up of 52.2 months, the addition of S-1 improved 5-year iDFS to 86.9% vs. 81.6% (HR 0.63, p = 0.0003), with primarily grade 3 neutropenia (~ 8%) and diarrhea (~ 2%) [7]. A post-hoc subgroup analysis demonstrated particularly strong benefit in patients fitting MonarchE eligibility (1–3 ALNs with high-risk features), with HR 0.47 (95% CI 0.29–0.74) [7].

### Clinical interpretation

Both MonarchE and POTENT confirm that augmenting endocrine therapy with either abemaciclib or S-1 significantly reduces recurrence risk in appropriate HR + /HER2– patients. MonarchE focuses on a strict high-risk cohort, reinforced by molecular markers like Ki 67, while POTENT includes a broader population, with risk defined by clinical-pathologic features. MonarchE’s CDK4/6 inhibition suits biologically aggressive disease, showing rapid and sustained benefit. In contrast, S-1 offers a cost-effective, tolerable option beneficial in intermediate-risk populations—especially where CDK4/6 inhibitors may not be accessible.

#### HER2-positive breast cancer treatment (trastuzumab and pertuzumab)

Trastuzumab (Herceptin®) and pertuzumab (Perjeta®) are established therapies for HER2-positive breast cancer. These monoclonal antibodies target the HER2 receptor, inducing apoptosis in tumor cells. The NeoSphere trial showed that the combination of trastuzumab and pertuzumab with chemotherapy increased the pathological complete response (pCR) rate in HER2-positive breast cancer patients [8].

In HER2-positive breast cancer, regular imaging is essential for assessing the therapeutic response. Specifically, MRI plays an important role in tracking tumor size and evaluating lymph node involvement. Achieving pCR is a significant factor in determining the success of treatment, and post-treatment imaging is essential in evaluating the presence of residual disease.

#### Immunotherapy and the role of tumor-infiltrating lymphocytes (TILs)

The development of immunotherapy, particularly immune checkpoint inhibitors (PD-1/PD-L1 inhibitors), has become a promising strategy in breast cancer treatment. Pembrolizumab (KEYTRUDA®) blocks the PD-1 receptor, activating tumor-specific cytotoxic T cells and inducing antitumor effects. The effectiveness of immunotherapy is closely linked to the presence of tumor-infiltrating lymphocytes (TILs), with higher TIL scores often correlating with better responses to chemotherapy and HER2-targeted therapies.

TILs are a key marker of immune response in the tumor microenvironment, and their presence can predict the effectiveness of treatment. Imaging techniques such as MRI and PET/CT can indirectly assess TILs and monitor changes in the tumor microenvironment. These imaging modalities allow for real-time tracking of treatment effects, helping to identify patients who are most likely to benefit from immunotherapy [9–13].

#### BRCA mutations and the role of poly ADP-ribose polymerase (PARP) inhibitors (olaparib)

For patients with BRCA mutation-positive breast cancer, PARP inhibitors like olaparib offer an effective therapeutic option. PARP is an enzyme involved in DNA repair, and in the absence of functional BRCA genes, PARP inhibition causes DNA damage that leads to cell death. This mechanism is particularly effective in patients with BRCA mutation-positive and HER2-negative breast cancer [14].

Imaging plays a key role in tracking the response to PARP inhibitors in breast cancer treatment. CT and MRI can be used to monitor tumor size and any structural changes that may indicate therapeutic success or failure. This is critical in determining the optimal treatment course for patients with BRCA mutations.

#### The tumor immune cycle and tumor microenvironment

The tumor immune cycle and the tumor microenvironment (TME) are critical factors influencing cancer progression and response to treatment. Tumor cells produce VEGF (vascular endothelial growth factor), which suppresses T-cell infiltration into the tumor. Additionally, tumor-associated fibroblasts (CAFs) and regulatory T cells (Tregs) create an immunosuppressive environment that supports tumor growth [15].

As immunotherapy continues to evolve, imaging plays an increasingly significant role in assessing changes in the tumor microenvironment. MRI and PET/CT are essential for monitoring these changes and providing valuable data to guide treatment decisions. For example, advanced MRI sequences (e.g., diffusion-weighted imaging) and novel PET tracers are being investigated to non-invasively map immune cell infiltration and activation [16, 17], helping to identify patients who are most likely to benefit from immunotherapy. Understanding the tumor microenvironment and its response to therapy is pivotal for improving outcomes and optimizing treatment strategies [18].

### Therapeutic approaches and imaging in clinical practice by subtype

#### Luminal subtype

Patients with Luminal A and B subtypes benefit from endocrine therapies, such as aromatase inhibitors, and targeted agents like CDK4/6 inhibitors (e.g., abemaciclib). The MonarchE trial demonstrated improved invasive disease-free survival (iDFS) in high-risk early-stage breast cancer patients receiving adjuvant abemaciclib [4, 5]. This finding emphasizes the importance of personalized treatment strategies for Luminal subtypes, where tailoring therapy based on the molecular profile of the tumor can lead to more effective outcomes.

The integration of imaging in the management of Luminal subtypes is critical. Pre-treatment imaging with mammography, ultrasound, and MRI can provide important insights into the extent of the disease, including lymph node involvement, tumor size, and the presence of any vascular or local tissue invasion. These findings guide the decision to administer neoadjuvant or adjuvant therapy. Post-treatment imaging further assists in evaluating the therapeutic response, detecting potential residual disease, and determining the need for further interventions such as radiation or extended hormonal therapy. The MonarchE trial, in particular, highlighted how imaging not only supports clinical decisions but also enhances the predictive accuracy of treatment strategies, ensuring that high-risk patients receive the most appropriate therapy [4, 5].

#### HER2-positive subtype

HER2-targeted therapies, such as trastuzumab and pertuzumab, have dramatically improved the outcomes for patients with HER2-positive breast cancer. Studies, such as NeoSphere, emphasize the predictive value of achieving pathological complete response (pCR) and its association with improved event-free survival (EFS) [8]. This finding is important since pCR is a reliable indicator of long-term survival, and imaging helps assess if this outcome has been achieved. MRI and CT scans are invaluable tools in monitoring the response to neoadjuvant HER2-targeted therapies, allowing clinicians to track the tumor’s size and assess lymph node involvement. The resolution of these imaging markers after treatment is often predictive of pCR, which can lead to tailored therapy decisions, such as discontinuation or continuation of HER2-targeted therapies [8].

#### Triple-negative breast cancer (TNBC)

Triple-Negative Breast Cancer (TNBC) is known for its aggressive nature and poor prognosis. However, the advent of immunotherapy, particularly immune checkpoint inhibitors like pembrolizumab, has provided new treatment options. The KEYNOTE-522 trial demonstrated that combining pembrolizumab with chemotherapy significantly improves both pCR rates and event-free survival in TNBC patients [9]. This combination therapy represents a paradigm shift, especially for patients with no other therapeutic options due to the lack of targetable receptors such as estrogen, progesterone, or HER2.

Imaging is indispensable for initial staging, therapy selection, and longitudinal evaluation in TNBC. Pre-treatment imaging helps in accurate staging, identifying the extent of the disease, and determining which patients may benefit from immunotherapy. Tumor-infiltrating lymphocytes (TILs), a biomarker for response to immunotherapy, can often be assessed through imaging-based techniques, especially in combination with biopsy and histological evaluations. Post-treatment imaging is crucial in detecting residual disease, and it can guide decisions about the need for further rounds of chemotherapy, immunotherapy, or clinical trials. As immunotherapy becomes more widespread in the treatment of TNBC, there will be an increasing need for better imaging techniques to monitor immune-related adverse events (irAEs), such as pneumonitis or colitis. FDG PET/CT is particularly sensitive in detecting these inflammatory complications, sometimes preceding CT abnormalities. These adverse effects can present similarly to cancer recurrence on imaging, making it crucial for clinicians to distinguish between the two.

## Case studies

### Case 1: luminal subtype

**Fig. 1:**
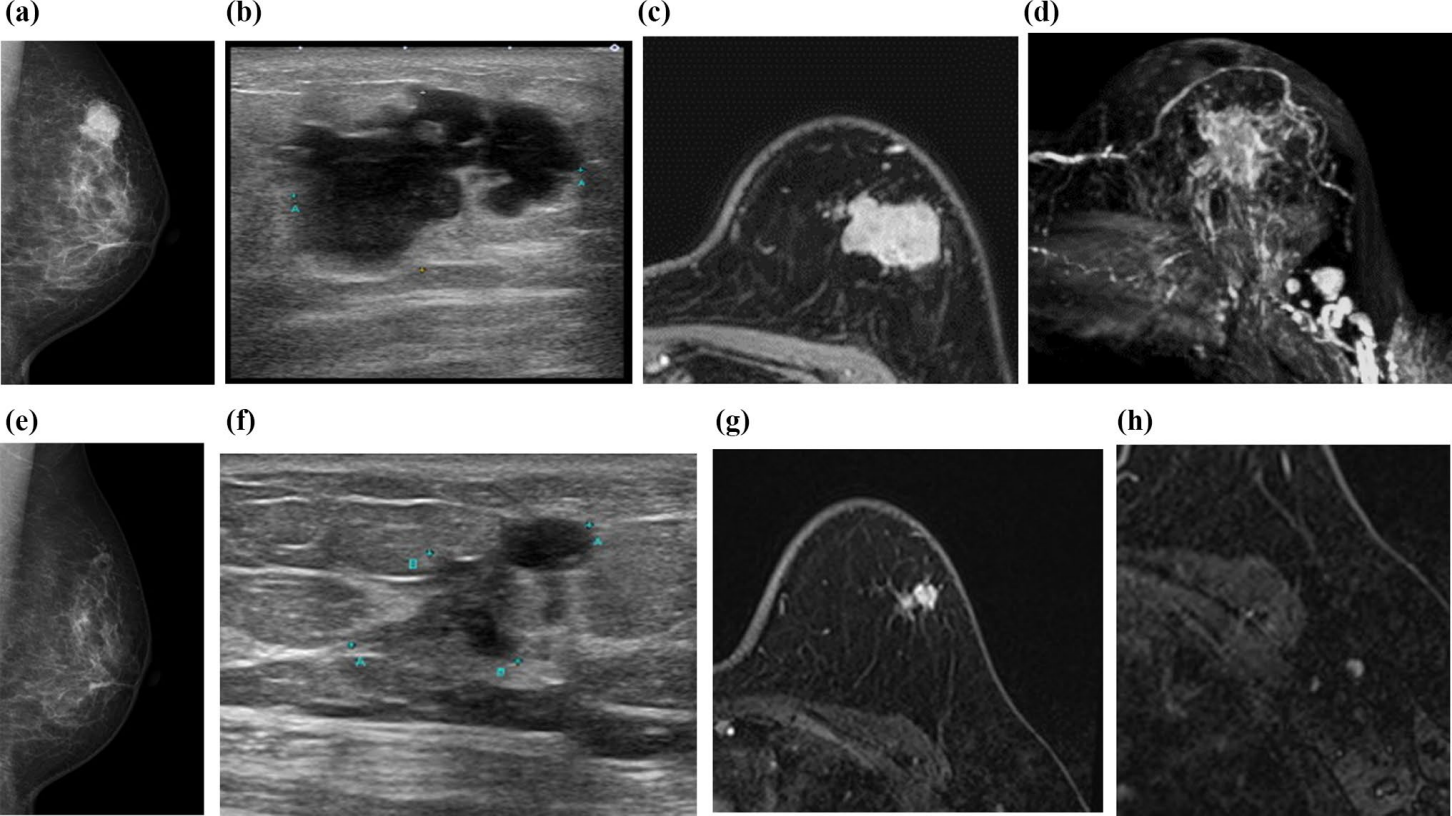
Pre- and post-treatment imaging of a woman in her 50s with ER-positive, HER2-negative breast cancer (Case 1). **a**–**d** Pretreatment imaging: **a** Mammography of the left breast, **b** Ultrasonography of the left breast, **c** Initial phase of dynamic contrast-enhanced (DCE) MRI, **d** MIP image of DCE MRI. A patient in her 50 s presented with a 3-cm tumor in the left breast and six metastatic lymph nodes. Mammography revealed an irregularly shaped, high-density mass with microlobulated margins in the left upper quadrant (**a**). Ultrasonography demonstrated a hypoechoic lobulated mass without posterior enhancement (**b**). The initial phase of DCE MRI showed an irregularly shaped mass with rapid homogeneous enhancement and metastatic lymphadenopathy in the left axilla (**c**, **d**). These findings were consistent with axillary lymph node metastasis involving four or more nodes, thereby meeting the eligibility criteria of the MonarchE trial. **e**–**h** Post-treatment imaging following neoadjuvant chemotherapy (FEC and DTX): **e** Mammography of the left breast, **f** Ultrasonography of the left breast, **g** Initial phase of DCE MRI, **h** Delayed phase of DCE MRI. Post-neoadjuvant chemotherapy imaging revealed significant tumor shrinkage and no residual lymphadenopathy (**e**–**h**). The scans demonstrated a complete or near-complete response, leading to continuation of CDK4/6 inhibition in the adjuvant setting (post-surgery) and resulting in long-term disease control

A 50-year-old patient with ER-positive, HER2-negative breast cancer presented with a 3 cm tumor and approximately six metastatic axillary lymph nodes (Fig. 1a–d), which corresponds to axillary lymph node metastasis involving four or more nodes, thereby fulfilling the eligibility criteria of the MonarchE trial.

The patient underwent neoadjuvant chemotherapy (FEC followed by DTX), followed by endocrine therapy combined with a CDK4/6 inhibitor. Posttreatment imaging (post-neoadjuvant therapy) revealed significant tumor shrinkage, no residual lymphadenopathy (Fig. 1e–h), and an overall response that led to long-term disease control.

This case illustrates how imaging can be used not only to monitor therapeutic response but also to guide clinical decisions regarding the continuation of therapy. The patient’s post-neoadjuvant imaging demonstrated a complete or near-complete response, and following surgery, the patient initiated endocrine therapy combined with a CDK4/6 inhibitor, which resulted in sustained long-term disease control. (This case was not part of a sponsor-initiated trial, and institutional approval and patient consent were obtained for publication.)

### Case 2: HER2-positive subtype

**Fig. 2:**
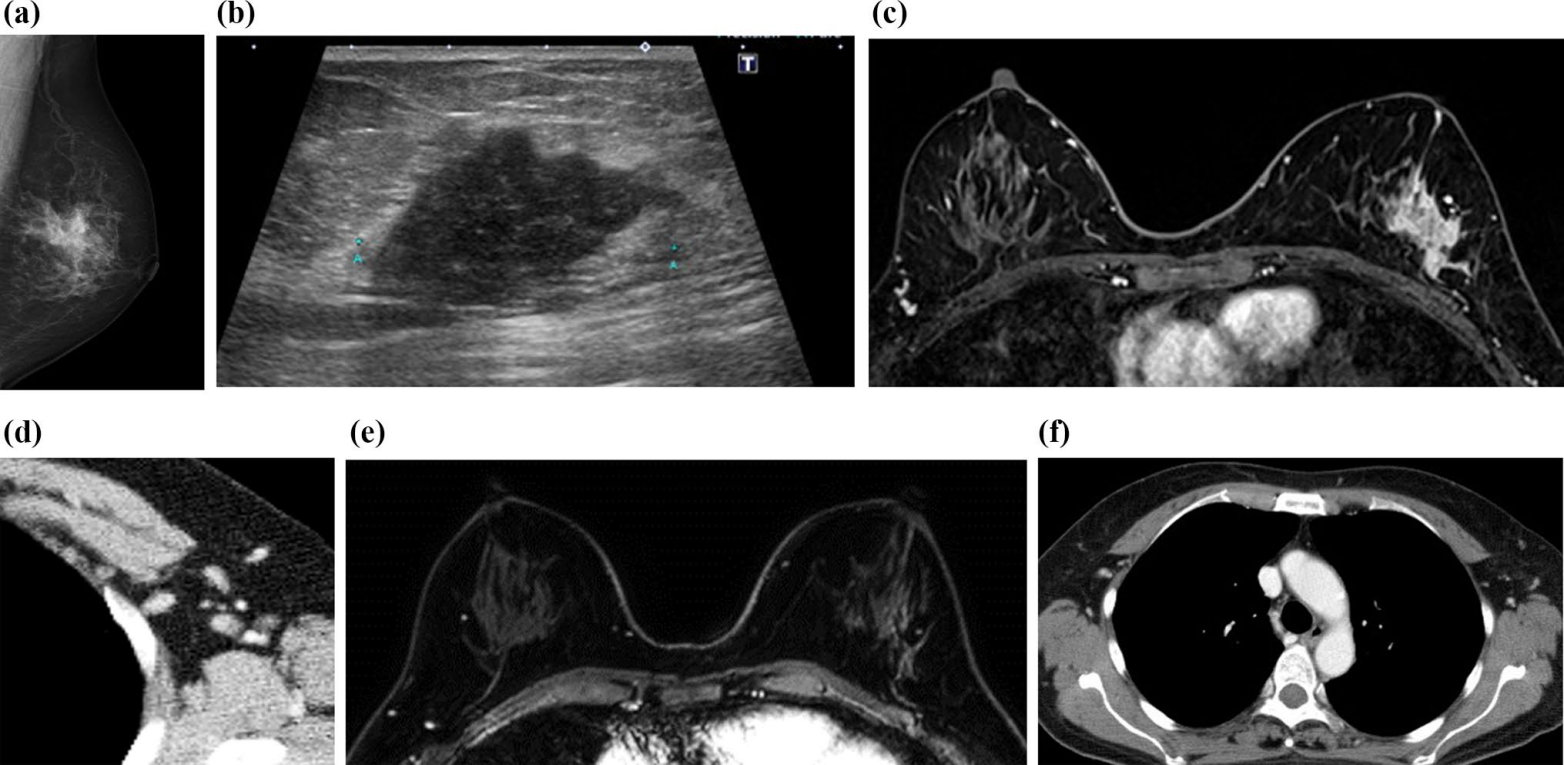
Pre- and post-treatment imaging of a woman in her 50s with HER2-positive breast cancer (Case 2). **a**–**d** Pretreatment imaging: **a** Mammography of the left breast, **b** Ultrasonography of the left breast, **c** Initial phase of dynamic DCE MRI, **d** Contrast-enhanced CT. Pretreatment imaging revealed a 4-cm tumor in the left breast with axillary lymph node involvement (**a**–**d**). **e**–**f** Posttreatment imaging following trastuzumab and pertuzumab in combination with chemotherapy: **e** Initial phase of dynamic contrast-enhanced (DCE) MRI, **f** Contrast-enhanced CT. Posttreatment MRI demonstrated no residual invasive carcinoma and no evidence of residual lymphadenopathy (**e**, **f**). The patient achieved pCR, validated by pathology, which correlated with the complete response seen on imaging

A patient in her 50s with HER2-positive breast cancer was treated with trastuzumab and pertuzumab in combination with chemotherapy. Pretreatment imaging revealed a 4-cm tumor in the left breast with axillary lymph node involvement (Fig. 2a–d).

Post-treatment MRI demonstrated no residual invasive carcinoma or lymphadenopathy (Fig. 2e, f). The patient achieved pCR, which was correlated with imaging findings of a complete response, and continued HER2-targeted maintenance therapy. In this case, imaging served as both a diagnostic tool and a means of assessing the efficacy of HER2-targeted therapies. The imaging finding of a complete response, which correlated with pathological pCR, allowed for a tailored treatment approach, avoiding unnecessary escalation of therapy while still maintaining a high level of surveillance for recurrence.

### Case 3: TNBC subtype

**Fig. 3:**
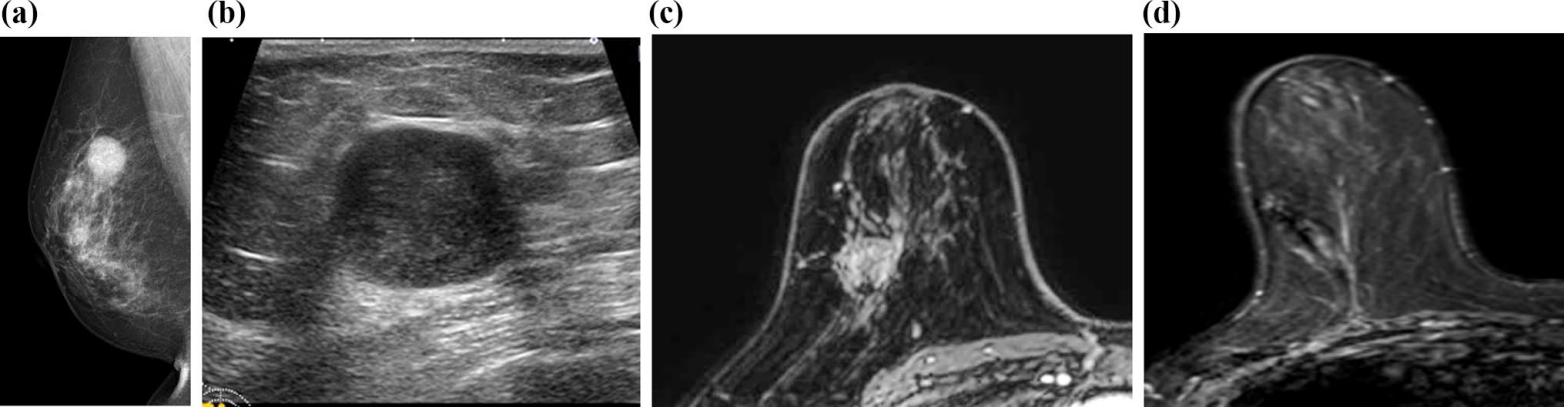
Pre- and post-treatment imaging of a partient in her 70s with TNBC (Case 3). **a**–**c **Pretreatment imaging: **a** Mammography of the right breast, **b** Ultrasonography of the right breast, **c** Initial phase of dynamic DCE MRI. Pre-treatment imaging revealed a well-defined, round primary tumor in her right breast (**a**–**c**) with two metastatic lymph nodes (not shown). **d** Post-treatment imaging following neoadjuvant chemotherapy combined with pembrolizumab: Post-treatment scans revealed marked tumor regression, and the patient achieved near-pCR status on DCE MRI (**d**)

A 70-year-old patient with TNBC underwent neoadjuvant chemotherapy combined with pembrolizumab. Imaging before treatment showed a large primary tumor in the right breast (Fig. 3a–c) with two metastatic axillary lymph nodes.

Post-treatment scans revealed marked tumor regression (Fig. 3d), and the patient achieved near-pCR status with improved overall prognosis. This case highlights the transformative potential of immunotherapy in TNBC. Imaging was crucial in monitoring the tumor’s response to the combined chemotherapy-immunotherapy regimen and demonstrating the effectiveness of pembrolizumab in achieving significant tumor shrinkage, a finding that directly influenced the course of treatment.

### Adverse effects and imaging

**Fig. 4:**
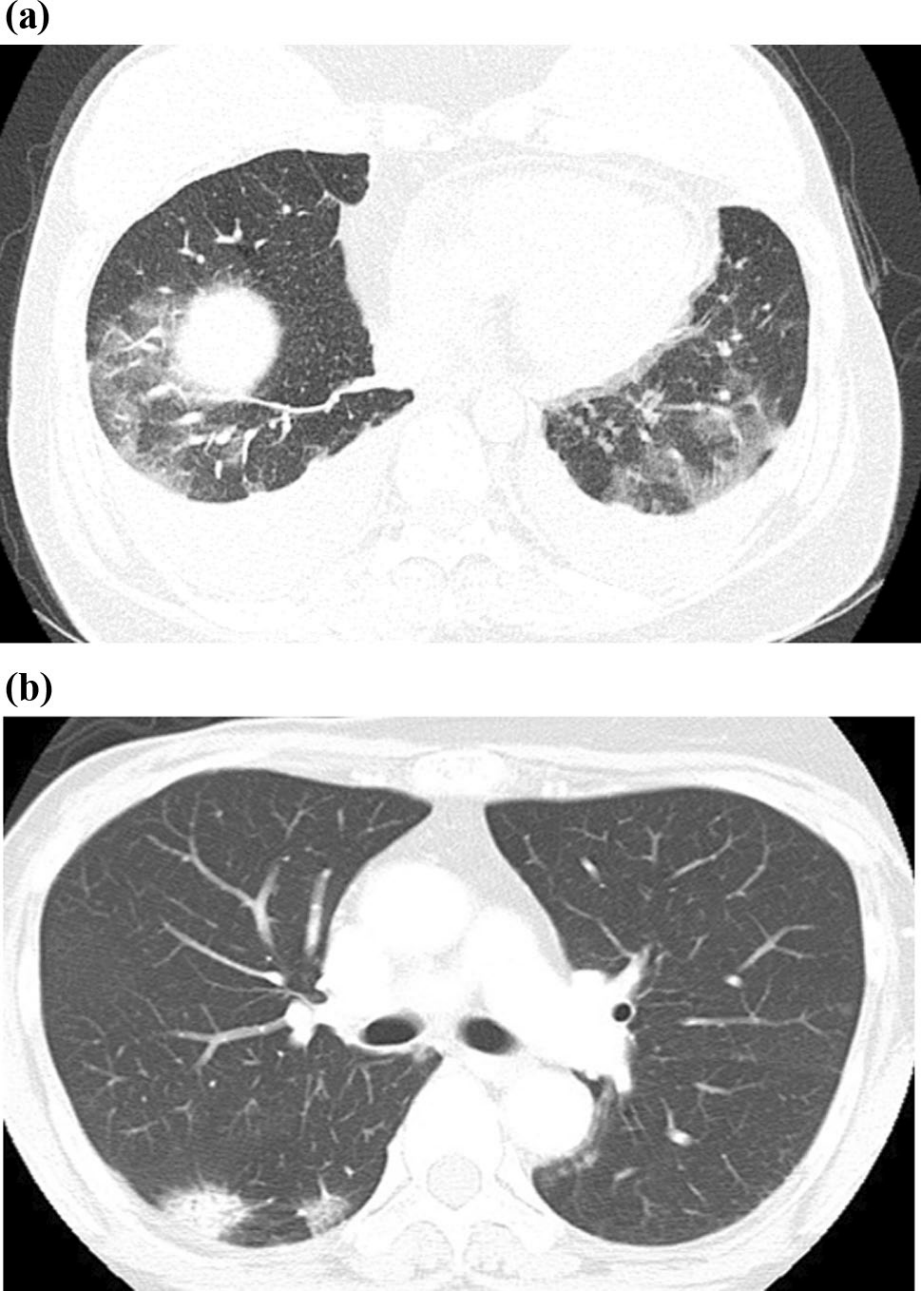
**a**, **b** Interstitial Lung Disease (ILD) in Two Patients Undergoing Systemic Therapy. **a** A woman in her 40s who underwent neoadjuvant chemotherapy (FEC followed by DTX) followed by endocrine therapy combined with a CDK4/6 inhibitor. She presented with follow-up CT findings suggesting ILD. **b** A different woman in her 40s with multiple metastases who was undergoing combined therapy with a CDK4/6 inhibitor. She complained of a cough and shortness of breath, and a CT scan revealed ILD. CT scans are essential for early detection of ILD, often identifying characteristic findings such as ground-glass opacities (**a**) or an organizing pneumonia pattern (**b**)

Adverse effects (AEs) related to cancer treatments, particularly those induced by CDK4/6 inhibitors and immune checkpoint inhibitors, represent a growing challenge. Drug-induced interstitial lung disease (ILD) is one of the most concerning complications, especially given the use of these therapies in combination with other agents. Imaging modalities like CT scans are essential for early detection of ILD, often identifying characteristic findings such as ground-glass opacities (Fig. 4a) or organizing pneumonia (OP) patterns (Fig. 4b) [19]. In a Japanese post-marketing study of breast cancer patients treated with abemaciclib, CT findings of ILD typically showed faint ground-glass opacities, OP–like consolidation, or diffuse alveolar damage (DAD) patterns; among these, DAD was strongly associated with poor outcomes. Older age, pre-existing interstitial changes, and poor performance status were additional predictors of fatal cases [20].

These findings can help clinicians make timely interventions, such as adjusting the dosage or discontinuing the therapy. The earlier that ILD is detected, the more effective management strategies can be in preventing irreversible lung damage.

This is particularly critical with antibody–drug conjugates such as trastuzumab deruxtecan, which has a known high frequency and severity of ILD. FDG PET/CT is also highly sensitive for detecting immune-related adverse events, such as pneumonitis or dermatitis. Diffuse pulmonary uptake or superficial skin uptake on PET may precede CT abnormalities, allowing for early detection [21].

**Fig. 5:**
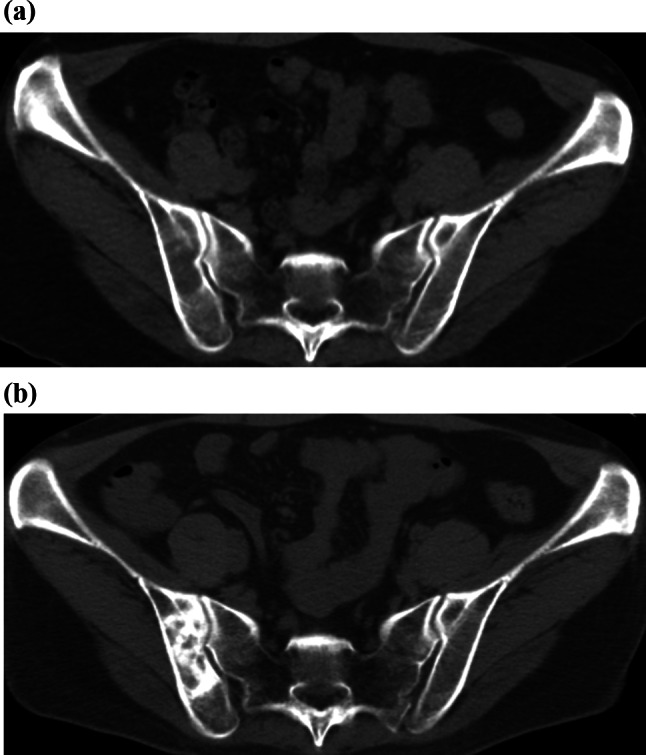
**a**,** b** A patient in her 50s receiving denosumab (anti-RANKL antibody) for bone metastases demonstrated, after six months of therapy, marked sclerosis in a previously inconspicuous osteolytic lesion of the right ilium (**b**, compared to baseline **a**), which should not be misinterpreted as new sclerotic metastases

Additionally, bone-related adverse effects, such as osteosclerotic changes linked to treatments like denosumab, are detectable through imaging (Fig. 5a, b).

Recognizing these changes early can prevent complications like fractures or bone pain, which may significantly affect the patient’s quality of life. Moreover, distinguishing between treatment-related bone changes and disease progression is crucial for making informed decisions about continuing or altering the treatment regimen [22, 23].

**Fig. 6:**
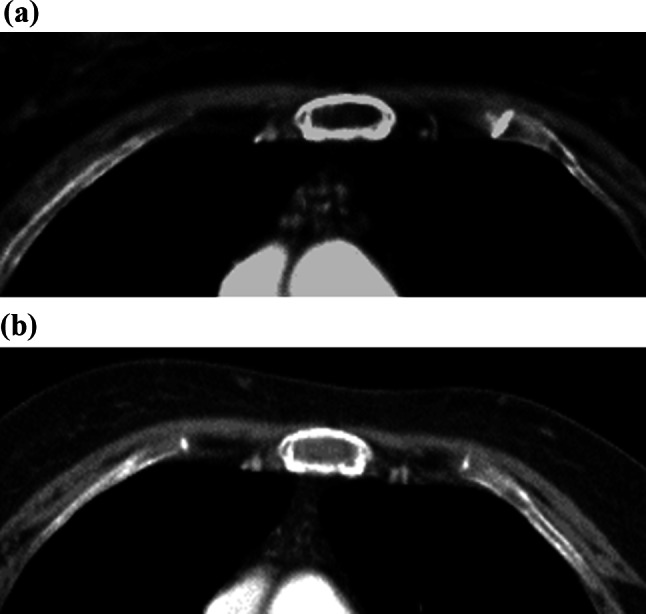
**a**, **b** A woman in her 70s receiving G-CSF for chemotherapy-induced neutropenia. CT demonstrates that the fatty marrow in the sternum (**a**, baseline) has changed to soft-tissue–like attenuation (**b**, post-G-CSF), representing hematopoietic marrow reconversion from yellow to red marrow. Caution is required to avoid misinterpretation as metastatic disease

G-CSF administration may induce notable changes in bone marrow appearance. CT can show the fatty marrow with soft-tissue attenuation (Fig. 6a, b), while MRI demonstrates corresponding low signal intensity on T1-weighted images and contrast enhancement.

These findings are indicative of hematopoietic marrow reconversion from yellow to red marrow. Careful review of the patient’s clinical history is essential to avoid misinterpretation as metastatic disease. These marrow changes are often much more clearly visualized on FDG PET/CT, typically showing diffuse, intense bone marrow uptake throughout the skeleton [24, 25].

### Endocrine therapy-related findings

Endocrine therapies, while generally well-tolerated, can induce specific imaging findings. Tamoxifen, for example, is associated with an increased risk of tamoxifen-induced fatty liver (hepatic steatosis) or hepatotoxicity. It can also occasionally cause the enlargement of uterine leiomyomas. Furthermore, tamoxifen is known to cause endometrial hyperplasia and an increased risk of endometrial cancer, necessitating regular gynecological surveillance with imaging and clinical evaluation [26–28].

### Interpretation of small lesions after therapy

Evaluating small residual or new lesions after systemic therapy requires careful consideration of the treatment context. After cytotoxic or HER2-targeted therapy, small, shrinking lesions in the contralateral breast or distant organs may indicate additional sites of malignancy that are responding to treatment. In contrast, during endocrine therapy, benign lesions (such as small fibroadenomas) may also exhibit shrinkage, and these must be distinguished from malignant lesions. While DCE-MRI and DWI are informative for larger lesions, the quantification of micro-lesions is significantly challenged by technical limitations, including low signal-to-noise ratio (SNR) in small voxels and susceptibility to patient motion, which compromise their reliability. Therefore, a multi-modality approach focusing on high functional and molecular specificity is essential for the definitive characterization of minute lesions. The high functional sensitivity of PET imaging, despite its own limitations in spatial resolution for sub-centimeter lesions, is highly valuable. The presence or absence of high FDG uptake on PET/CT is highly informative in distinguishing active malignant disease from benign changes. Furthermore, novel molecular PET tracers (e.g., [^18^F]FES PET/CT for estrogen receptor status or [^18^F]FLT for proliferation) are under investigation to provide size-independent characterization based on specific biological activity [29, 30].

## Conclusion

Advancements in drug therapies and imaging diagnostics have revolutionized breast cancer management, enabling personalized treatment strategies tailored to the biological characteristics of each tumor. The integration of imaging in treatment planning, monitoring, and management of adverse events enhances the precision of care and optimizes patient outcomes. As new therapies, such as immune checkpoint inhibitors, continue to emerge, further studies should explore how imaging can be utilized in conjunction with biomarkers to refine treatment protocols and improve both survival rates and quality of life for breast cancer patients.

In conclusion, imaging has become indispensable in the era of precision medicine, not only for monitoring therapeutic responses but also for detecting and managing treatment-related complications. Further research will continue to enhance the synergy between advanced imaging technologies, including PET/CT, and novel therapeutic agents, providing new insights into personalized breast cancer care.
